# Pre-symptomatic treatment of spinal muscular atrophy: a strategic shift from concept to clinical practice

**DOI:** 10.1007/s12519-026-01039-0

**Published:** 2026-05-11

**Authors:** Shan-Shan Mao

**Affiliations:** Department of Neurology, Children’s Hospital, Zhejiang University School of Medicine, National Clinical Research Center for Children and Adolescents’ Health and Diseases, Hangzhou, 310052 China

The treatment of spinal muscular atrophy (SMA) has undergone a fundamental strategic transformation over the past decade, shifting from “symptom-driven intervention” to “intervention before irreversible motor neuron loss”. The core pathophysiology of SMA lies in the progressive degeneration and apoptosis of α-motor neurons in the anterior horn of the spinal cord and brainstem [1, 2]. The age of onset and clinical severity vary among different SMA phenotypes. Studies in severe cases, such as newborns and infants with SMA type I, have reported that 50%–70% of motor neurons have been permanently lost when symptoms manifest. This established the basic therapeutic principle that “time is motor neurons” or put another way time leads to progressive motor neuron loss [3]. Pre-symptomatic treatment with the aim of disease onset avoidance is no longer a theoretical concept but a practical choice based on current scientific evidence and clinical practice. This should allow affected children to reach a motor development level close to that of unaffected children of the same age. Key clinical trials and real-world practice confirm that patients receiving pre-symptomatic treatment achieve greater clinical benefits on motor function development and reach normal motor milestones [4–8]. International consensus has established pre-symptomatic treatment as a standard strategy for SMA [9] and recommended that disease-modifying therapy (DMT) should be initiated as soon as possible upon diagnosis via newborn screening (NBS) [9, 10]. In this context, SMA–NBS is expanding worldwide. Indeed, by early 2024, at least 33 countries across the Americas, Europe, Oceania and Asia, including the United States, Germany, Australia and Japan, have established national or large‑scale regional screening programs. These programs cover approximately 7% of newborns globally and consequently pre-symptomatic treatment for SMA [11]. However, very few regions in China have launched pilot studies on NBS and pre-symptomatic treatment [8, 12]. In general, this strategic shift has transformed the core concern from “whether to treat” to “how to systematically achieve pre-symptomatic diagnosis and treatment”. This commentary discusses issues that constitute a framework for implementation.

## What is the current status of and challenges to implementation of pre-symptomatic treatment for pediatric SMA?

The first challenge is the identification of patients with SMA; routine NBS is recommended, as it is both effective and feasible [9, 10, 13]. Currently, a few European and American countries have incorporated SMA into national routine NBS programs. In China, NBS for SMA is not widespread, and many patients are diagnosed after the onset of clinical symptoms; this misses the optimal window for pre-symptomatic treatment. In countries without routine and widespread NBS (like China), SMA diagnosis is usually made via identification of a pathogenic survival motor neuron (SMN) 1 gene variant through genetic testing. This is performed during disease screening, prenatal diagnosis of blood relatives [14, 15], or during medical examination for other diseases [15], where there are no clinical symptoms of SMA. Collectively, this places higher demands on obstetricians and gynecologists for accurate prenatal counseling and an ability to identify pre-symptomatic SMA with clinical sensitivity.

Current NBS for SMA mainly detects the homozygous deletion of exon 7 in the *SMN1* gene using real‑time quantitative polymerase chain reaction performed on dried blood spots [9]. A complete diagnostic process should be initiated immediately as the deletion of *SMN1* is confirmed [9]. Positive screening results should be reported to both clinical specialists and patient’s caregivers on the same day. Initial assessment should include a confirmatory genetic test for *SMN1* deletion alongside *SMN2* (the main phenotypic modifier gene for SMA) copy number and a comprehensive clinical evaluation. This clinical evaluation should include a detailed neurological physical examination, neuro-electrophysiological testing, and multi-system functional assessment. This should be conducted concurrently and completed within two days of notification [9]. Initiation of DMT is recommended as soon as possible in children with positive confirmatory test results [9, 16]. For high-risk infants with two copies of the *SMN2* gene, the time window from screening to the initiation of treatment should be minimized as much as possible, imposing higher requirements on screening technology [9, 16]. Integrating *SMN2* copy number detection into the initial screening process can significantly shorten the time from positive screening to treatment decision-making and improve diagnostic efficiency.

Although three DMT drugs have been approved for marketing in some countries, their application in pre-symptomatic treatment is restricted by the absence of universal NBS. Overcoming these barriers requires highly coordinated medical and policy-level support, including an integrated testing process for the simultaneous acquisition of *SMN1* and *SMN2* genetic information. Moreover, a standardized referral pathway, establishing a rapid referral channel between screening laboratories and regional diagnostic and treatment centers, is needed along with baseline multi-system functional assessment within 48 hours of positive testing. In addition, a clinically supported family-centric informed decision-making process is essential [9].

## How can *SMN2* copy number and emerging biomarkers inform risk stratification and treatment timing in screen-positive infants?

In addition to monitoring motor milestone development and performing precise neurological examinations to maintain the “pre-symptomatic status”, several biomarkers may provide essential objective evidence for distinct clinical phases of the disease. Specifically, *SMN2* copy number serves as the primary biomarker for guiding the decision to initiate treatment, while other emerging biomarkers, such as compound muscle action potential (CMAP) amplitude and neurofilament light chain (NfL), could be utilized to monitor disease progression and treatment response throughout the whole course.

As the main genetic modifier of the SMA phenotype, *SMN2* copy number plays a guiding role in treatment decision-making [17]. Patients with two copies of *SMN2*, with an extremely high risk of developing severe type I SMA, should be regarded as a “neurological emergency”, and the core of treatment decision-making is “racing against time” [17]. Patients with three copies of *SMN2* exhibit significant clinical heterogeneity, even displaying a mild phenotype; in these cases, pre-symptomatic treatment can significantly improve long-term motor milestone achievements [17]. International consensus also advocates for an active early treatment for these patients with two or three copies of *SMN2* [9]. The management of asymptomatic patients with *SMN2* ≥ four copies is the most controversial. However, recent registry studies suggest that most patients with four copies of *SMN2* will still develop significant symptoms in early childhood or throughout their lives [18], emphasizing the need for an active management strategy.

Asymptomatic SMA infants may already show a sub-clinical reduction in CMAP amplitude, providing objective evidence for early intervention [19, 20]. For patients with *SMN2* ≥ three copies who have not yet initiated treatment [20], dynamic monitoring of trends in CMAP change can serve as a basis for treatment decision-making. A progressive decrease of over 30% within 6 months can be taken as a trigger for treatment [20].

NfL and phosphorylated heavy chain are sensitive biomarkers of neuronal injury [19, 21]. Especially, the levels of NfL in cerebrospinal fluid and blood of SMA patients significantly correlate with disease severity [21]. Elevated NfL levels can be detected at the pre-symptomatic stage, and a decrease after treatment is associated with clinical improvement [22]. This is, therefore, an effective tool for monitoring treatment response.

## Providing lifelong care for children receiving pre-symptomatic SMA treatment requires interdisciplinary collaboration

Patients receiving pre-symptomatic treatment for SMA need lifelong care. This is a collaboration between doctors, medical systems, caregivers, and governments. Comprehensive and accurate understanding by multiple cooperating parties that SMA is a “treatable but not yet curable” disease will better help consolidate and sustain such a lifelong care model. Collectively this approach will maximize the benefits of pre-symptomatic treatment.

Nusinersen, onasemnogene abeparvovec, and risdiplam have been approved as DMT drugs for SMA, with preliminary evidence in pre-symptomatic treatment [4–8]. Eight-year follow-up data from the NURTURE study showed that most pre-symptomatic children receiving nusinersen treatment reached the expected developmental milestones within the World Health Organization developmental milestone window with a good safety profile [5]. In the SPRINT trial, asymptomatic infants treated with onasemnogene abeparvovec achieved motor development trajectories close to normal [7]. In addition, preliminary data from the RAINBOWFISH study also confirmed the favorable efficacy of risdiplam in pre-symptomatic children [6]. When selecting DMT, the medical team needs to comprehensively integrate the child’s clinical characteristics (age, weight, *SMN2* copy number, and multi-system function), therapeutic properties of different potential drugs (duration of action, dosing frequency and route of administration), family factors (treatment preferences, caregiving capacity, and economic status), and healthcare system factors (drug accessibility, local medical technical capacity, and medical insurance reimbursement policies) into decision-making [9, 17]. For high-risk infants with two copies of *SMN2*, the timeliness of treatment initiation is more critical than the optimization of drug selection [17]. When multiple regimens are applicable, a doctor–patient-shared decision-making model is recommended to formulate personalized treatment pathways.

Given that SMA is a complex neurodegenerative disease with potential multi-system dysfunction, and pre-symptomatic intervention initiates lifelong therapeutic journey spanning multiple life stages from infancy to adulthood, the whole-process clinical monitoring of multi-system functions in pre-symptomatic patients needs to be integrated into the standard management [9], rather than relying solely on DMT, to capture potential multi-system complications and adjust interventions timely throughout long-term follow-up. Effective whole-course management requires multidisciplinary team integration, including pediatric neurologists, rehabilitation physicians, physical therapists, pulmonologists, dietitians, orthopedists, and psychologists [17, 23]. As the child grows, adult neurologists, occupational therapists, and social workers need to be incorporated [24]. Key success factors include regular multidisciplinary consultations, clear case management systems, shared medical record systems, and active family participation. A systematic pediatric-to-adult healthcare transition plan should be formulated, usually initiated in early adolescence, to ensure both continuity and coordination of medical services [23, 24]. The focus of follow-ups should be flexibly adjusted with age [17, 23–26]. In the first 2–3 years of life, the focus is on the development of motor function, monitoring of treatment safety, and complication screening, with assessments every 3–6 months. From preschool to adolescence, the emphasis is on functional independence, educational preparation, and social adaptation, with assessments every 6–12 months, while also monitoring multi-system function and psychosocial development. In addition, doctor–patient communication should adopt targeted strategies, such as clearly explaining disease mechanisms and the necessity of early intervention through intuitive metaphors, using charts of clinical trials and real-world data to visually demonstrate the long-term prognostic differences between pre-symptomatic and post-onset treatments. There should be frank discussions of practical challenges, such as economic burdens, joint formulation of feasible management plans, linking support resources, and providing continuous social and psychological support. The classical doctor role needs to transform into “health partner”, working with caregivers to develop personalized and sustainable management strategies.

In China, promoting pre-symptomatic treatment also requires multi-level and systematic policy interventions. This includes incorporating NBS into national public health programs, improving the three-level prevention framework, and integrating SMA management into the existing maternal and child health and rare disease prevention and control systems. Simultaneously, conducting health economics research in line with national conditions to evaluate long-term social and economic benefits is critical.

## Summary and future perspectives

To achieve a true therapeutic revolution for SMA, it is necessary to promote a fundamental transformation in disease management paradigms with pre-symptomatic treatment as the core. This requires not only a shift in the treatment time window but also a systematic reconstruction of disease management concepts and systems. Specifically, this includes establishing a national NBS network with rapid diagnostic channels, developing precise stratification strategies based on *SMN2* copy number and biomarkers, constructing a lifelong multidisciplinary management system, and forming a cost-effective, equitable, and accessible policy framework. Despite multiple challenges in SMA diagnosis and treatment, significant opportunities for future progress remain. Next-generation treatment options (safer vectors and *SMN*-independent strategies), intelligent decision support systems, and positioning SMA as a ‘model disease manageable through a three-level prevention system’ will provide a strategic template for the management of other rare diseases.

## Data Availability

No datasets were generated or analyzed for this commentary.
